# A Defect-Free Vertical-Cavity GaAs-Based Nanowire Laser on Silicon Emitting at the Telecom O‑Band

**DOI:** 10.1021/acs.nanolett.5c03702

**Published:** 2025-09-19

**Authors:** Cem Doganlar, Paul Schmiedeke, Markus Döblinger, Jona Zöllner, Benjamin Haubmann, Severin Reitberger, Knut Müller-Caspary, Jonathan J. Finley, Gregor Koblmüller

**Affiliations:** † Walter Schottky Institute, TUM School of Natural Sciences, Technical University of Munich, 85748 Garching, Germany; ‡ Department of Chemistry and Center for NanoScience, Ludwig-Maximilians-University Munich, 81377 Munich, Germany; § Institute of Physics and Astronomy, Technical University Berlin, 10623 Berlin, Germany

**Keywords:** III−V nanowire lasers, telecom band, monolithic integration on Si, multiple quantum wells, PL spectroscopy, transmission electron microscopy, nanobeam electron diffraction

## Abstract

Telecom-band vertical-cavity nanowire (NW) lasers are promising integrated light sources for silicon (Si) photonics applications but have remained elusive within the important GaAs materials system. Here, we demonstrate the direct site-selective integration of a vertical-cavity GaAs-based NW laser on Si that exhibits lasing emission in its as-grown geometry at the telecom O-band (∼1.3 μm). This achievement relies on an advanced NW heterostructure using an InGaAs/InAlGaAs multiple quantum well (MQW) active gain region coaxially integrated on a vertical GaAsSb NW core with high Sb content to minimize strain energy. Consequently, uniform composition throughout the entire MQW and minimal strain (<1.3 ± 0.2%) with no extended defects are verified by scanning transmission electron microscopy and nanobeam electron diffraction. Single-mode lasing is consistently observed for a range of operation temperatures under optical pumping with lasing thresholds as low as 160 μJ/cm^2^. Mode-dependent threshold gain analyses reveal further that a high-order transverse mode is responsible for lasing.

## Graphical abstract

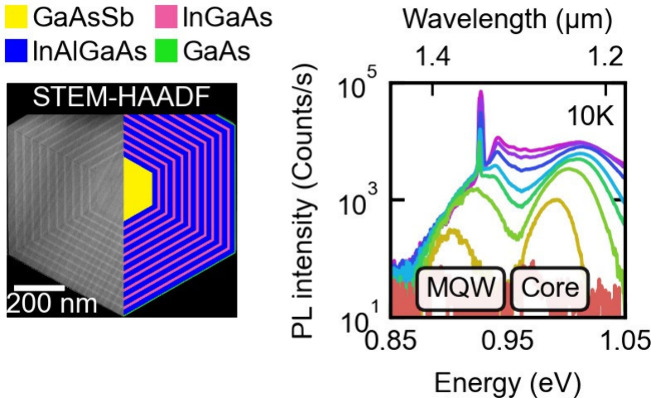

Semiconductor nanowires (NWs) hold great potential as compact and cost-efficient nanoscale lasers because they offer both a gain medium and a microcavity for efficient optical feedback all in one structure.^1,2^ Furthermore, their submicron-size footprint presents an ideal pathway for highly integrated photonic systems with low power consumption,^3–5^ and several examples of III–V-based NW lasers on various platforms have been demonstrated.^4–12^ In this regard, telecom-band NW lasers that are integrated on photonic circuits, especially Si photonic integrated circuits (PICs), are particularly appealing because they are promising on-chip coherent light sources for high-speed optical communication and computing.^3,8–12^

Several studies have demonstrated these application potentials so far, given that NW lasers can be site-selectively integrated on Si and a silicon-on-insulator (SOI) platform using scalable epitaxial growth processes.^8,9,12–15^ Consequently, monolithic, on-chip integrated NW lasers have enabled also straightforward emission coupling schemes to Si waveguides and PICs, shown for, e.g., single III–V (GaAs) NW^8^ and nanopillar (InP) lasers,^15^ as well as 1D-nanobeam-cavity (InGaAs NW-array) lasers.^9^ Nanobeam-cavity lasers rely on extended external cavities;^9,14^ hence, the smallest possible cavities that meet the ultimate size-scaling requirements are only offered in single, vertical-cavity NW lasers. Such compact monolithically integrated NW lasers have, however, not yet been reported at telecom-band wavelengths for the GaAs-on-Si platform, pointing to some outstanding challenges in realizing vertical-cavity NW lasers in this materials system.

Clearly, bulk-like GaAs NW lasers^16,17^ have a band-gap energy (1.42 eV) too high to access Si-transparent wavelengths, necessitating gain media in the form of either coaxial quantum wells (QWs) or axial quantum disks (QDs) embedded in the GaAs-on-Si platform. Here, both InGaAs and GaAsSb QWs/QDs have been explored as active regions,^6,18–20^ but the high In/Sb contents (>35%) required to reach telecom-band emission have caused too high cumulative strains and mismatch-related defects that deteriorate the optical properties. GaAs/InGaAs- and GaAs/GaAsSb-based NW lasers have therefore shown emission mostly at short wavelengths of ∼0.8–1.1 μm.^6,8,13,16–20^ One way to mitigate the internal strain is to use strain-compensating buffer layers, shown in, e.g., coaxial InGaAs/GaAs MQW NW lasers.^21^ However, such lasers have not been realized in as-grown, vertical-cavity geometry, pointing to substantial losses at the Si substrate interface. Another substantial factor affecting the lasing performance is the crystal polytypism and associated defects like twinning and planar defects seen in typical GaAs NW lasers.^17,22^ These defects cause nonradiative recombination^23^ and, thereby, contribute to large internal optical losses, further hampering the net optical gain.

Here, we demonstrate a new and defect-free GaAs/InGaAs-based NW-laser heterostructure that surpasses previous limitations to realize telecom-band lasing from single NWs at the O-band (1.3 μm), all in its direct, vertical-cavity form on Si. Figure 1a,b displays a schematic illustration and correponding scanning electron microscopy (SEM) image of the monolithically integrated single vertical-cavity NW laser on a SiO_2_-masked Si (111) substrate. The NWs are grown by selective area molecular beam epitaxy (MBE) using a combination of self-catalyzed vapor–liquid–solid (VLS) growth for the NW core (GaAsSb)^24,25^ and vapor–solid (VS) growth for the coaxial InGaAs/InAlGaAs MQW active region. Details about all respective growth parameters and the SiO_2_/Si pattern design (using a 15 nm thin SiO_2_-mask layer) are described in the Supporting Information. As seen in the SEM image, NW is perfectly vertically aligned on the substrate, confirming the direct epitaxial growth on Si.^24^ Furthermore, the length of the NW laser measures approximately 7.4 μm, providing a sufficiently long cavity thanks to the meticulously developed GaAsSb core growth in our recent work.^24,25^

**Figure 1:**
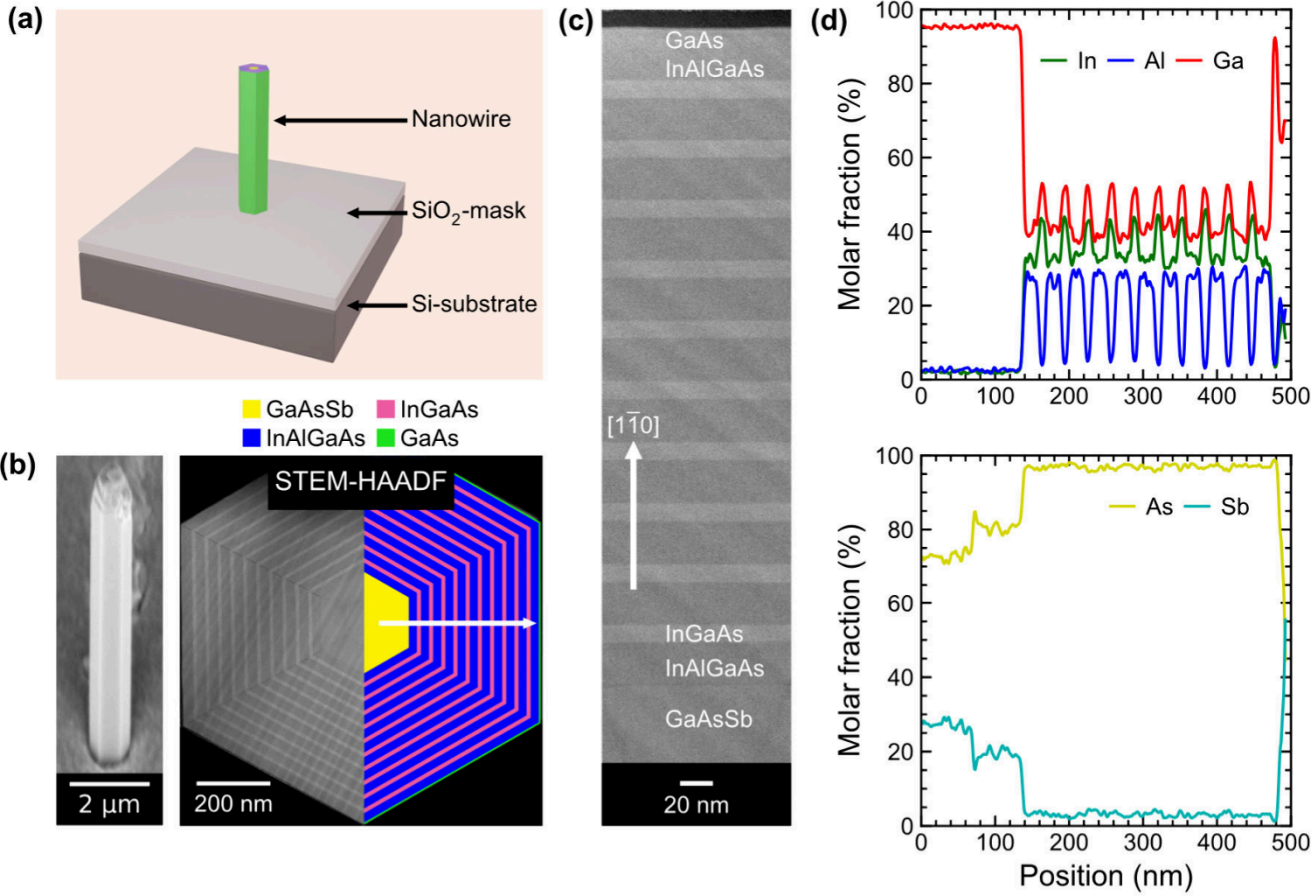
Structural and compositional properties of GaAsSb–InGaAs/InAlGaAs NW lasers. (a) Schematic illustration of a monolithically grown single NW laser on a prepatterned Si (111) substrate (using a 15 nm thin SiO_2_-mask layer). (b) SEM image of a single NW laser and corresponding FIB-prepared cross-sectional STEM-HAADF micrograph illustrating the uniform MQW heterostructure. The right-hand-side schematic illustrates the material systems that are used for the individual layers. (c) STEM image of the 10-period MQW structure along one of the major ⟨11̅0⟩ orientations. (d) EDXS line scans showing the elemental distribution of the Group III (top) and Group V (bottom) species, recorded along ⟨11̅0⟩ from center to surface (see the arrow in the STEM-HAADF image in part b).

Statistical analysis performed on several NWs suggests only a small variation in length (∼7.3 ± 0.2 μm), while the total NW diameter varies between 988 and 1133 nm (see the Supporting Information for additional SEM images). This variation is mainly induced by the GaAsSb core diameters of ∼300–450 nm, as confirmed by reference growths,^24^ while the thickness variation of the MQW region is negligible. Due to the Sb-rich GaAsSb core growth, no stacking defects are observed along the entire cavity length, as verified by previous transmission electron microscopy (TEM) experiments.^24^

High-angle annular dark field scanning transmission electron microscopy (HAADF-STEM) and energy-dispersive X-ray spectroscopy (EDXS) are used to map the coaxial MQW heterostructure via a radial cross-sectional, focused ion beam (FIB) cut through the center region of the NW. As shown in Figure 1b, the NW displays a highly symmetric hexagonal shape, and the core and MQW regions, consisting of 10 periods of InGaAs QWs and InAlGaAs barriers, can be well distinguished by their atomic number (*Z*) contrast. The image is overlaid by a schematic on the right depicting the individual layers of the core–multishell heterostructure. Figure 1c shows a high-magnification image of the MQW layer sequence recorded along one of the six major ⟨11̅0⟩ facets, confirming the uniform QW thickness and interface abruptness across the entire radial direction. Higher-resolution STEM images of the InGaAs/InAlGaAs interface region (see the Supporting Information) further evidence a perfectly ordered crystal lattice with no visible defects.

The QW and barrier thicknesses analyzed statistically across all six side facets are 9.5 (±0.3) and 23.5 (±0.2) nm, respectively. The EDXS-measured alloy composition recorded along one of the major ⟨11̅0⟩ facets of the NW is presented in Figure 1d for both the Group III (Al, Ga, and In) and Group V (As and Sb) elements. It can be seen that the GaAsSb NW core consists of two regions with slightly different Sb contents (∼30% in the inner region and ∼20% in the outer region), consistent with earlier observations.^25^ In contrast, the MQW structure exhibits a uniform composition, with high In content in the QWs (In_0.44_Ga_0.56_As) and sufficiently lower In content (In_0.32_Ga_0.40_Al_0.28_As) in the barriers. All layer compositions agree with the nominal values within the experimental error (±5%) of the EDXS analysis (see the Supporting Information). The compositional uniformity of the MQW structure was further examined along the length of the NW using confocal microphotoluminescence (μ-PL) spectroscopy (see the Supporting Information). The spatially resolved PL spectra (recorded at 10 K and low pump fluence) evidence a consistent PL peak energy (i.e., 0.9 eV) of the spontaneous emission of the MQW active region throughout the entire NW length, confirming the high uniformity of the heterostructure along the cavity length.

Individual vertical-cavity NW lasers were then probed in their as-grown geometry on a Si substrate under pulsed optical excitation in our μ-PL setup (see details for excitation conditions in the Supporting Information). Figure 2a shows pump-fluence-dependent spectra recorded at 10 K of a typical NW laser. At the lowest pump fluence (*P*), the spectrum is characterized by two broad spontaneous emission (SE) peaks centered around 0.89 and 0.97 eV. The low-energy peak is attributed to the MQW active region, while the high-energy peak stems from the GaAsSb core. The observation of emission from both the core and MQW is not surprising given that the entire heterostructure is excited at sufficiently large energy (1.59 eV) and that the core and MQW regions are separated by an intermediate barrier (see the Supporting Information). The emission energy of the core also agrees well with the previous data of bulk GaAsSb NW lasers when grown under the same conditions.^25^ This suggests that the coaxial InGaAs/InAlGaAs MQW heterostructure is nearly lattice-matched to the GaAsSb core and exerts only minimal strain (see also the 4D-NBED data below). When the pump fluence is increased, both peaks shift to higher energy (about ∼50 meV each, for pump fluences raised by 2 orders of magnitude), which is indicative of band filling. At a sufficiently high pump fluence (*P* = 216 μJ/cm^2^), a singular sharp peak emerges from the MQW region at 0.928 eV, corresponding to an emission wavelength of 1.34 μm (telecom O-band). This peak shows nonlinear growth characteristic of amplified spontaneous emission and then transitions to linear scaling above this threshold with much larger intensity compared to the SE background, which is characteristic for lasing. At this point, also no further blue-shift with pump fluence is observed in the SE background, suggesting gain clamping.^26^

**Figure 2:**
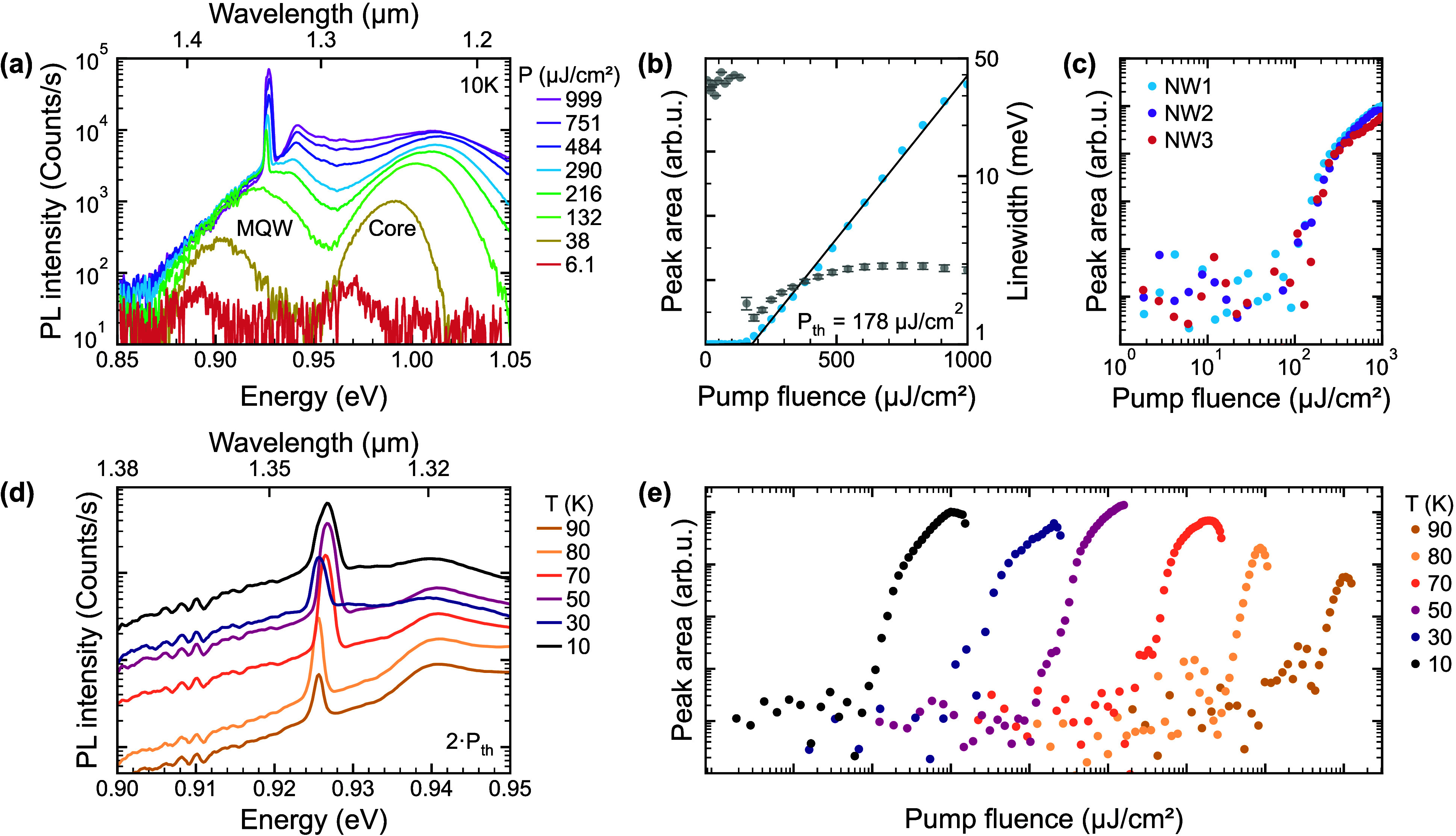
Lasing properties of as-grown NW lasers on Si. (a) Pump-fluence-dependent PL spectra of a single GaAsSb–InGaAs/InAlGaAs NW laser probed in its vertically integrated geometry on Si at 10 K. (b and c) Light input–light output (L–L) curves of the MQW active region in linear and logarithmic representation, illustrating the lasing threshold (linear fit in part b) and s-shape characteristics (c), including data from two additional NW lasers. The line-width narrowing with increasing pump fluence is also shown in part b with gray data points. (d and e) Lasing emission and corresponding L–L curves at different lattice temperatures, offset by a factor of 10 along the *x* axis between the respective temperatures. The lasing peaks shown in part d are for a fixed pump fluence at twice the lasing threshold (2·*P*_th_).

To shed more light on the lasing characteristics, we plot the integrated peak intensity and emission line width of the MQW emission from the same NW laser in Figure 2b as a function of the pump fluence. This plot clearly demonstrates the strong increase in intensity occurring at the lasing threshold fluence, which is 177.9 ± 9.0 μJ/cm^2^ as obtained from linear fitting of the data. Simultaneously, at the lasing threshold, we observe strong line-width narrowing, i.e., the line width of the MQW emission drops from 38.5 meV for *P* < 178 μJ/cm^2^ to 1.7 meV when exceeding the lasing threshold. The lasing behavior is further manifested in the typical s-shape of the input–output characteristics seen on a double-logarithmic scale in Figure 2b. Here, for comparison, we also plot the input–output curves of two other NW lasers grown on the same Si substrate, which show nearly identical behavior. Corresponding spectra are shown in the Supporting Information, verifying the single-mode lasing emission at the same wavelength (1.34 μm). The lasing thresholds of the additional lasers are found to be 160 (±8) μJ/cm^2^ and 209 (±20) μJ/cm^2^, respectively. This relatively small variation in the threshold among the different devices highlights the good homogeneity due to selective area growth. The lasing threshold values are also on par with those reported for other near-IR and long-wavelength III–V NW lasers (see the Supporting Information).

The single-mode lasing characteristics are further maintained over a range of temperatures, as demonstrated in Figure 2d. The spectra show representative lasing emission recorded up to 90 K at a constant pump fluence above the lasing threshold (at ∼2·*P*_th_), where the main lasing peak is normalized to the background emission. Characteristic input–output curves at the different temperatures are presented in Figure 2e (note that the L–L curves are offset by a factor of 10 along the *x* axis for clarity). Telecom-band lasing is consistently observed for all lattice temperatures, with the lasing peak remaining centered around 1.340 (±0.003) μm. The corresponding lasing threshold is found to increase for this NW laser from 178 (±9.0) μJ/cm^2^ (10 K) to 408 (±95) μJ/cm^2^ at 70 K, illustrating the typical thermal broadening of the gain spectrum with increasing temperature.^17,18^ The temperature-dependent lasing of the other two NW lasers exhibits very similar trends (see the Supporting Information). Room temperature lasing was not yet observed; however, in the discussion below, we provide clear guidelines on how this can be realized.

Overall, the observation of lasing with a threshold in the sub-mJ/cm^2^ range for directly monolithically integrated telecom-band NW lasers is fairly surprising at first glance, given the underlying integration design. Note that the vertical-cavity NW lasers employ only a thin SiO_2_-mask layer (∼15 nm thin), which limits the modal reflectivity^17^ of typical low-order transverse optical modes (≪1%; see the Supporting Information). Hence, to understand the low-threshold single-mode lasing emission in our vertical-cavity NW lasers, we anticipate two factors being crucial: (i) the very high material quality (material gain) of the active MQW region with minimal internal losses and (ii) the presence of a higher-order mode inducing lasing.^27^

To examine this hypothesis, we performed finite-difference time-domain (FDTD) calculations of the threshold gain for a number of different transverse modes propagating in our 7.4-μm-long vertical-cavity NW laser that is placed on a 15 nm thin SiO_2_ layer with a hollow mask opening (50 nm diameter), through which the NW laser is anchored to the Si substrate. The vertical-cavity NW laser is further surrounded by an ∼1-μm-thick parasitic III–V layer at its bottom interface separated by ∼150 nm from the NW, as measured by cross-sectional SEM (see the Supporting Information). In our analysis, we emphasize the longitudinal cavity modes because the vertical-cavity NW laser represents a distinct FP-type modal cavity, as seen by the FP resonances in the PL spectra (Figure 2a and the Supporting Information). The results are shown in Figure 3 for the most prominent modes, along with their respective electric-field-intensity profiles. Additional data, particularly of the modal reflectivity, effective refractive index, and optical confinement factor, are presented in the Supporting Information. To account for any variation in the cavity dimensions, the threshold gain data are plotted here as a function of the experimentally observed changes in the GaAsSb core diameter.

**Figure 3:**
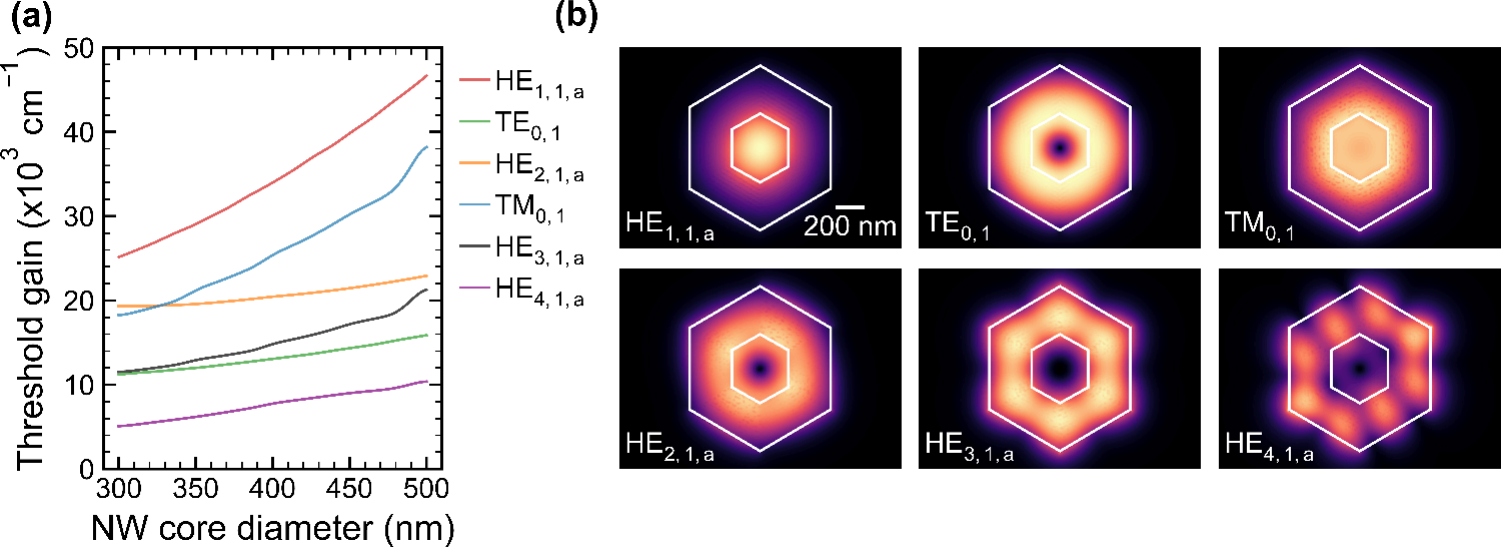
Threshold gain and optical mode analysis. (a) Calculated threshold gain as a function of the NW core diameter at an emission wavelength of 1.34 μm, under otherwise fixed thickness of the MQW region. The plot summarizes the threshold gain dependence for the most prominent optical modes (for twin modes, only the *a*-component is shown), illustrating the lowest threshold gain for the high-order HE_4,1,a_ mode. (b) Corresponding energy distributions of the respective modes shown for a fixed total diameter of 400 nm.

For the lowest-order (HE_1,1,a/b_) modes, the energy distribution is mainly concentrated at the center axis of the NW core (Figure 3b); hence, their interaction with the gain medium (MQW region) is low.^13^ The threshold gain of these modes increases with rising core diameter, due to a decrease in mode confinement that results from the respective increase in the ratio of the gain medium versus total NW diameter (see the Supporting Information). Crucially, the HE_1,1,a/b_ modes leak heavily into the substrate through the hollow mask opening at the bottom facet, resulting in very low reflectivity and hence a very high threshold gain (>30000 cm^–1^). Similar behavior is also observed for the TM_0,1_ mode and, to some extent, also for the HE_2,1,a/b_ modes because their energy distributions are also centered in close proximity to the NW core. On the other hand, the TE_0,1_ mode shows a threshold gain 2 times lower than that of the HE_1,1,a/b_ modes. This is because the TE_0,1_ mode is azimuthally polarized and offers enhanced interaction with the MQW gain region, improving mode confinement.^10^ Its energy distribution is also closer to the periphery of the NW such that the reflectivity is less impacted by the bottom facet. Still, its modal reflectivity is relatively poor (<1%) to allow for low-threshold lasing. Instead, high-order modes, such as the HE_4,1,a_ mode presented in Figure 3, provide a much better optical feedback. This mode results in higher modal reflectivity of up to 3–4% despite the low-index-contrast bottom interface (Supporting Information Video). Consequently, the threshold gain is substantially lower (∼4000 cm^–1^) and relatively insensitive to variations in the NW diameter. This mode further supports strong light–matter interaction in the NW cavity due to its high group refractive index (see the Supporting Information), making it the most likely mode for the single-mode lasing observed in the vertical-cavity NW lasers. The threshold gain of the HE_4,1,a_ mode can be further lowered by increasing modal reflectivity, e.g., by increasing the SiO_2_ layer thickness (see ), as will be investigated in the future.

To address the second important factor supporting our hypothesis, we verify the high quality of the MQW gain medium by performing 4D-STEM-based nanobeam electron diffraction (NBED) experiments. These measurements, as detailed in the Supporting Information, allow quantitative maps of the strain distribution throughout the entire structure and, thus, provide information about the presence or absence of mismatch-induced strain relaxation and related defects.^28,29^ Hereby, the lattice variation parallel and perpendicular to all six radial growth facets is mapped because these indicate whether the QWs are fully strained to each other or whether plastic relaxation is potentially present locally (see the Supporting Information). Data from two of these directions are shown in Figure 4, specifically along the [202̅] and [12̅1] directions, for which we denote $\kappa_{xx}=\frac{d_{20\mathrm{2̅}}}{d_{20\mathrm{2̅}}^{0}}$ and $\kappa_{yy}=\frac{d_{1\mathrm{2̅}1}}{d_{1\mathrm{2̅}1}^{0}}$ as the relative lattice plane spacing calculated with respect to the average value in the NW core (analogous data of the other four directions are presented in the Supporting Information). For the horizontally aligned hexagon segments, they describe lattice plane variations perpendicular and parallel to the radial growth facets (101̅) and (1̅01) in the cross section. Parts a and b of Figure 4 display 2D maps of κ_*xx*_ and κ_*yy*_. In the horizontal segments, κ_*xx*_ shows strong lattice variations, revealing fully strained growth in the radial growth direction (see the Supporting Information). In comparison to the inner core, the lattice of the QWs shows a pronounced radial expansion of approximately 1.5%, while the lattice of the buffer layers remains similar, and the outer core lattice is contracted, as expected from the different compositions according to Vegard’s law. In contrast, κ_*yy*_ always stays close to zero, indicating fully coherent interfaces without plastic relaxation and virtually the same lattice parameters of all segments along the interfaces. Further maps with κ_*xx*_ and κ_*yy*_ measured along the other directions ⟨202̅⟩ and ⟨12̅1⟩ show the same symmetry and similar magnitudes (see the Supporting Information). Parts c and d of Figure 4 shows line scans along the [101̅] direction through the horizontal segments (indicated by arrows in Figure 4a,b), quantitatively representing the radial and lateral lattice variations κ_*xx*_ (Figure 4c) and κ_*yy*_ (Figure 4d) at the radial growth facets (101̅) and (1̅01). The experimental data are compared to strain simulations with the *nextnano-3* software, using a 2D freestanding strain model for the NW heterostructure.^21^ In the simulations, the strain is evaluated by minimizing the total elastic energy density in the cross section of the NW on the basis of experimentally determined compositions. Because strain refers to the deformation of a fully relaxed cell (whereas the measurements use the NW core as reference), the *nextnano* results were converted to κ_*xx*_ and κ_*yy*_ as defined above, such that the experiment and theory in Figure 4c,d are directly comparable. The experimental data in Figure 4c show a radial lattice contraction of around 1% in the outer core, a negligible variation of the buffer layers, and an expansion of 1.3% in the QWs versus the buffer layers. The measured lattice variation κ_*yy*_ along the facets of the layers (Figure 4d) is small overall (<0.6%) and free of abrupt changes at interfaces between the layers. The simulated profiles agree quantitatively very well with the experimental results, confirming that the MQW NW structure exhibits coherent and strained interfaces without relaxation. The absence of strain relaxation is also manifested by the specular sidewall morphologies^20,21^ of the NW laser (cf. Figure 1b), further supporting the defect-free nature of the present NW-laser structure.

**Figure 4:**
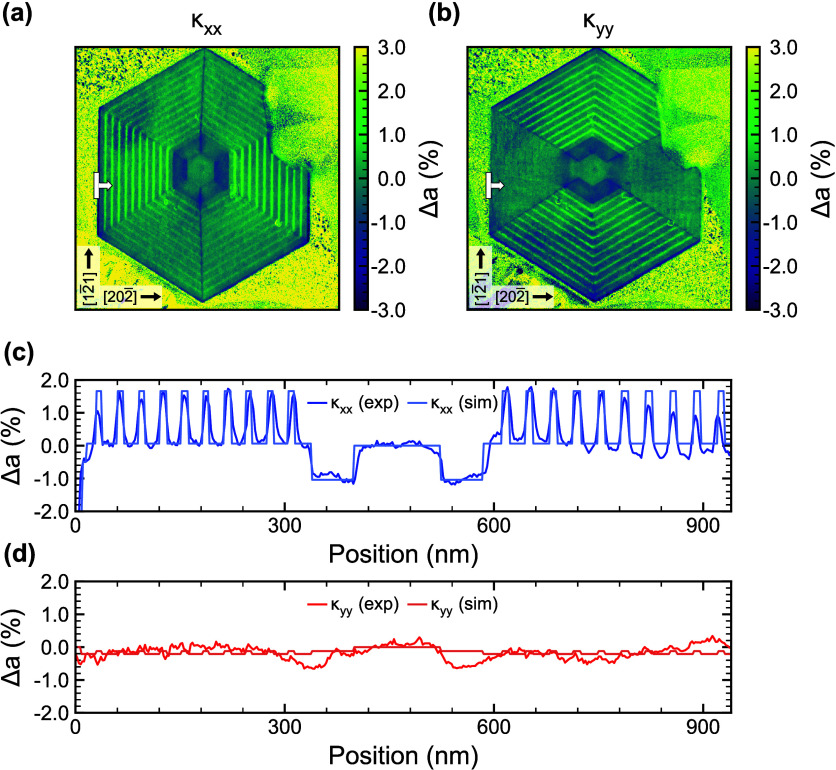
Analysis of the strain state of the NW-laser structure by NBED experiments and *nextnano* simulations. (a and b) Experimentally measured 2D maps of the relative lattice spacings, taking [202̅] and [12̅1] as the basis, yielding κ_*xx*_ and κ_*yy*_. (a) Lattice parameter variation in the growth direction for the horizontally aligned segments of the hexagonal cross section. (b) Homogeneity of the lateral lattice parameter indicating fully strained, coherent growth. (c and d) Corresponding line scans along the [101̅] direction through the horizontal hexagon segments (indicated by arrows in parts a and b), alongside their simulated counterparts. The measured κ curves are shown in blue and red, while the simulated κ curves are displayed in light blue and light red.

In summary, we demonstrated the first GaAs-based vertical-cavity NW laser directly integrated on Si with lasing emission in the important telecom O-band (∼1.3 μm). Single-mode lasing with a comparatively low threshold (<200 μJ/cm^2^) was established under optical pumping over a range of temperatures. This achievement relies on two essential factors: First, high-quality InGaAs/InAlGaAs MQW active regions embedded coaxially on a defect-free vertical GaAsSb-based NW core with minimal radial strain. Second, a high-order transverse mode mitigates the poor modal reflectivity of the low-index GaAs/Si interface to achieve lasing in its ultracompact vertical-cavity geometry. Further tuning the modal reflectivity, e.g., via high-contrast SiO_2_ interlayers with large thickness, will allow even greater control of the lasing threshold and temperature performance. These findings now open a clear route for establishing monolithically integrated, GaAs-based telecom-band NW lasers with ultracompact footprints on Si photonic circuits.

## Supplementary Material





## Data Availability

Data that support the findings of this study are available from the corresponding author upon reasonable request.

## Notes

Details of selective-area molecular beam epitaxy, additional SEM images, PL spectroscopy of additional NW lasers, FDTD threshold gain simulations, and details about STEM and NBED characterization and additional data (PDF)
Video of the evolution of mode propagation (MP4)
