# Supplementary material for: A Defect-Free Vertical-Cavity GaAs-Based Nanowire Laser on Silicon Emitting at the Telecom O‑Band
Source: Nano Lett. 2025 Sep 19;25(39):14377–83. doi: 10.1021/acs.nanolett.5c03702 (PMC12492396; doi:10.1021/acs.nanolett.5c03702)
Supplement: Supplementary file 1 [file nl5c03702_si_001.pdf]

Supporting Information for

A defect-free vertical-cavity GaAs-based nanowire laser on silicon emitting at the telecom O-band

Cem Doganlar[1,*], *Paul Schmiedeke*[1], *Markus Döblinger*[2], *Jona Zöllner*[1], *Benjamin Haubmann*[1], *Severin Reitberger*[1], *Knut Müller-Caspary*[2], *Jonathan J. Finley*[1], *Gregor Koblmüller*[1,3]

[1]Walter Schottky Institute, TUM School of Natural Sciences, Technical University of Munich, 85748 Garching, Germany

[2]Department of Chemistry and Center for NanoScience, Ludwig-Maximilians-University Munich, 81377 Munich, Germany

[3]Institute of Physics and Astronomy, Technical University Berlin, 10623 Berlin, Germany

*Corresponding authors.

E-mail: cem.doganlar@tum.de (C.D.); gregor.koblmueller@tu-berlin.de (G.K.)

S1. Selective-Area Molecular Beam Epitaxy (SAE)

The NW lasers in this work were grown by selective-area molecular beam epitaxy (MBE) in a fully position-controlled fashion on prepatterned 2-inch Si (111) substrates, covered by ≈20 nm-thick SiO_2 mask layer. Using electron beam lithography (EBL) and wet chemical etching (buffered hydrofluoric (HF) acid), patterns of circular hole opening arrays (d_{hole} = 50 nm) were created in the SiO_2 mask layer. The prepatterned hole arrays act as nucleation sites for self-catalyzed VLS NW growth, which results in GaAsSb NW cores with a length of 7.4 μm and a defect-free pure zincblende (ZB) crystal structure [1]. Here, the growth process was adapted from previous optimization of high aspect-ratio GaAsSb NWs using a multi-step growth process: first, Ga prewetting was performed for 2 min under a Ga-flux of 2.35 Å s^{-1} at a substrate temperature of 660 °C, to create Ga droplets for NW nucleation inside the hole openings. Subsequently, a short (~300-nm) GaAs stem was grown for 3 min under an As-BEP (beam equivalent pressure) of 4×10^{-6} mbar to realize NW nucleation with sufficient yield. After this the Ga-flux was ramped down to 0.4 Å s^{-1} to proceed with GaAsSb growth by adding Sb with a BEP = 1.6×10^{-6} mbar. To achieve the desired length of > 7 μm, the growth was adjusted by incrementally increasing the Ga-flux (in steps of 0.2 Å s^{-1}), in growth intervals of 30 min, i.e. raising the Ga-flux from 0.4 to 0.6 Å s^{-1}, and further to 0.8 Å s^{-1} and 1 Å s^{-1} (4-step growth process) [1]. After finishing the core growth, the axial growth is terminated by closing the Ga-shutter and crystallizing the Ga-droplet. This was then followed by reducing the substrate temperature to 360 °C and increasing As-BEP to 4.0×10^{-5} mbar for consecutive high-quality radial MQW growth on the NW sidewall facets. Here, a total number of 10 $In_xGa_{1-x}As$ QWs with quaternary $In_xAl_xGa_{1-2x}As$ barriers was grown. As for the growth parameters, an equal rate of In, Al and Ga-fluxes of 0.45 Å/s was taken, which was expected to result in nominally 20-nm thick $In_{0.33}Al_{0.33}Ga_{0.33}As$ quaternary barriers and 8-nm

thin $In_{0.5}Ga_{0.5}As$ QWs in-between. Finally, the structure was capped by 5 nm GaAs layer to prevent oxidation of the surface. A scanning electron microscopy (SEM) image of a typical as-grown field of NW lasers is shown in **Figure S1**, along with high-magnification images of two individual NWs from the same field. The images confirm the relatively high uniformity in their dimensions and their morphological properties, despite the observed low growth yield. The growth yield of self-catalyzed (Ga-mediated) GaAs NWs grown site-selectively on Si is known to be governed by the initial Ga droplet forming during nucleation [2-6]. In our case, prewetting of Ga was employed prior to nucleation [1], and this step is essential for determining the formation and morphology of Ga droplets that serve the subsequent VLS growth [2-5]. Here, droplet size plays a critical role in

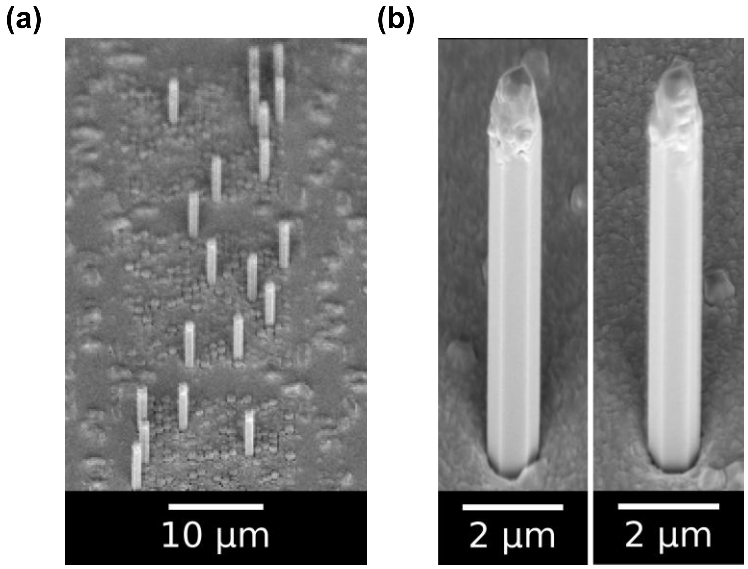

Figure S1. SEM images of NW lasers as-grown on the patterned Si (111) substrates. (a) Low-magnification image showing NWs with relatively uniform dimensions. (b) High-magnification images of individual NWs demonstrating further the very similar morphological properties.

this process, with larger droplets in bigger mask openings being more prone to crystallization and irregular morphology (through interaction with residual As in the growth environment), while

smaller droplets maintain the spherical liquid shape necessary for successful NW nucleation [6]. Precise control of prewetting conditions is therefore crucial, as improper droplet formation during this initial stage can drastically reduce the overall nanowire growth yield.

S2. Structural and Compositional Analysis

To analyze the structural integrity and compositional homogeneity of the coaxial MQW heterostructure across the NW, both spatially resolved micro-photoluminescence (µ-PL) spectroscopy and high-angle annular dark-field scanning transmission electron microscopy (STEM-HAADF) were performed. First, to demonstrate the overall homogeneity of the MQW active region across the entire NW laser structure, spatially resolved PL spectra were recorded on single NWs that were transferred to a Si support substrate. Details of the PL setup and experimental conditions are further provided below in Section S3. Hereby, spectra were taken in increments of 1 µm along the NW, from the base (position 1) all the way to the tip (position 6) at 10 K and low pump fluence of 3.7 µJ/cm^2 (spot size 2 µm). The low pump fluence ensures that only the spontaneous emission is probed, which best captures any variations in the homogeneity of the QWs. As shown in **Figure S2**, the PL spectra acquired at the different positions along the NW match quite well both in the peak intensity and position, which is centered near 0.90 eV, irrespective of the excitation position. This indicates an excellent compositional and structural uniformity along the length of the NW. Only near the NW base the spectral intensity is slightly weaker, which is expected because here the QW growth is shadowed by the parasitic layer forming on the growth substrate. In addition, the PL spectra show a slight peak deconvolution at such low pump power independent of excitation position. We believe that this may stem from minor

fluctuations in alloy composition within the QW or changes in carrier confinement induced by slight changes in the InAlGaAs barrier composition between the corner <112>B facets and the

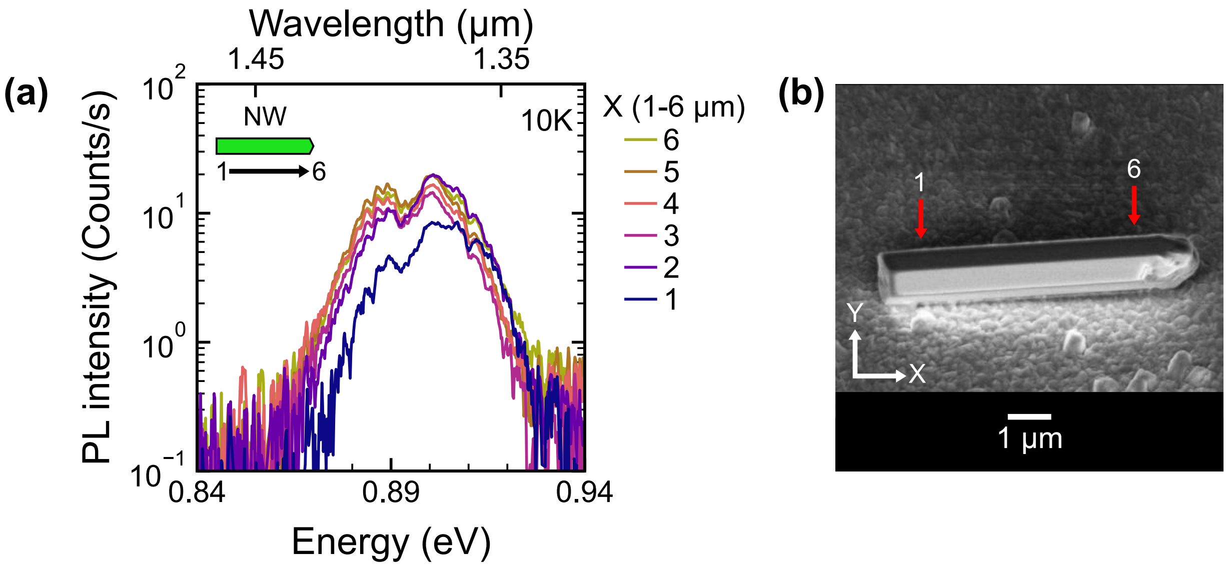

Figure S2. (a) Spatially resolved photoluminescence (PL) spectra of the MQW emission recorded at 10 K and low pump fluence (3.7 μJ/cm^2). Each spectrum was acquired at 1 μm intervals along the length of a single nanowire, shown in the SEM image (b).

main sidewall <110> facets (see STEM-HAADF images below in Figures S3-S5). However, these do not significantly impact the overall high quality and uniformity of the MQW heterostructure in the lasing structures, since only a singular spontaneous emission from the MQW is observed at higher pump fluences (cf. Fig. 2(a) and Fig. S6).

To further characterize the radial homogeneity of the MQW heterostructure, a cross-sectional lamella was prepared for STEM analysis using focused ion beam (FIB) milling. First, the material composition and thickness of each individual layer was investigated by energy dispersive X-ray Spectroscopy (EDXS) and HAADF-STEM). **Figures S3 and S4a** (below) show maps of the elemental distribution of In, Al, Ga and Sb and the atomic number (Z)-contrast across both the

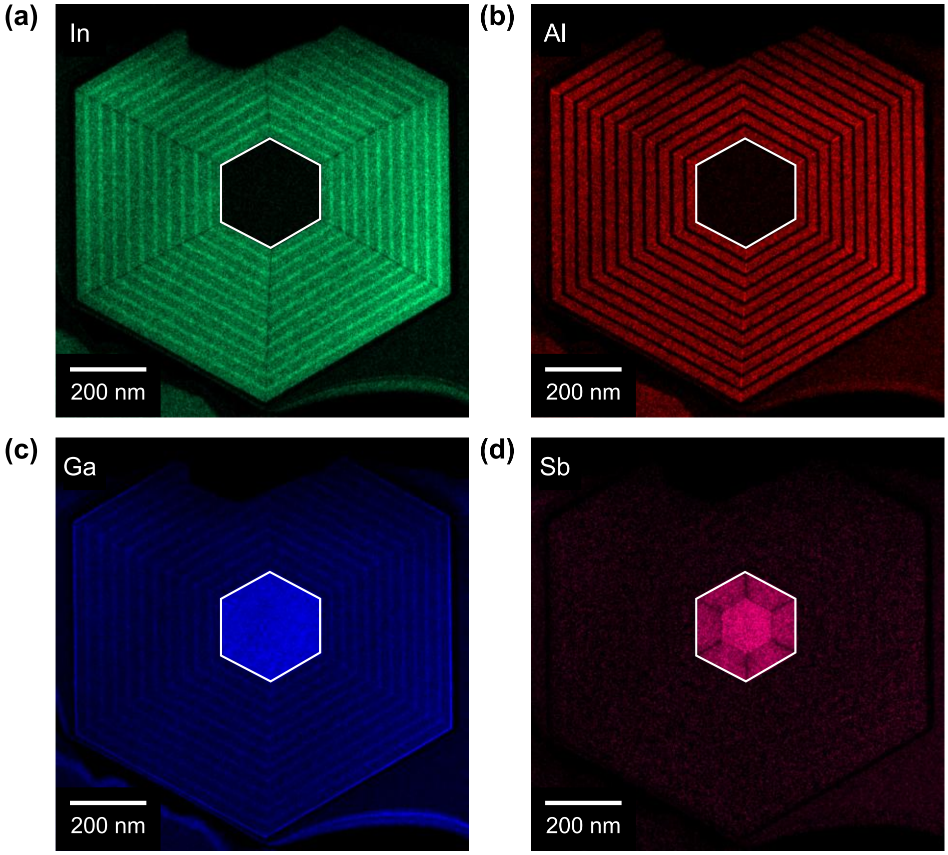

Figure S3: EDXS elemental maps of the MQW NW-laser structure in radial cross-section. The white hexagon demarcates the GaAsSb core region. From (a)-(d), the distribution of group-III elements In- (green), Al- (orange), Ga (blue) and group-V element of Sb (pink) are presented.

NW-core and MQW regions.

At a first glance, the typical symmetric hexagonal shape of NW can be clearly observed and the MQW structure appears to be homogeneous across all sidewall facets. Furthermore, in both **Figure S3(a,b)** and **Fig. S4(a)** the individual InGaAs QW layers can be clearly distinguished, i.e., as brighter regions due to atomic mass difference induced Z-contrast. From the STEM analysis, the thickness of the quantum well and barriers are found to be 9.5 (±0.3) nm and 23.5 (±0.2) nm, matching with the nominally expected values, with an experimental error less than 19%. Moreover, to confirm the homogeneity of the alloy composition in the active region, an EDXS line scan was

taken along another equivalent $\langle 1\bar{1}0 \rangle$ direction as that shown in the main text (orange arrow). **Figure S4**b and **Figure4**c show the corresponding elemental distributions of group-III and group-V species, respectively. Within the GaAsSb core, the Sb-content is slightly lower in the outer region (~20%), compared to the innermost section of the core, verifying our previous findings [1]. The composition of the InGaAs QWs and InAlGaAs barriers is, within the experimental error of EDXS (~3-5%), identical to the values identified along other equivalent $\langle 1\bar{1}0 \rangle$ direction (cf. main text Fig. 1(d)). This confirms the overall homogeneity of the MQW structure across the various sidewall facets.

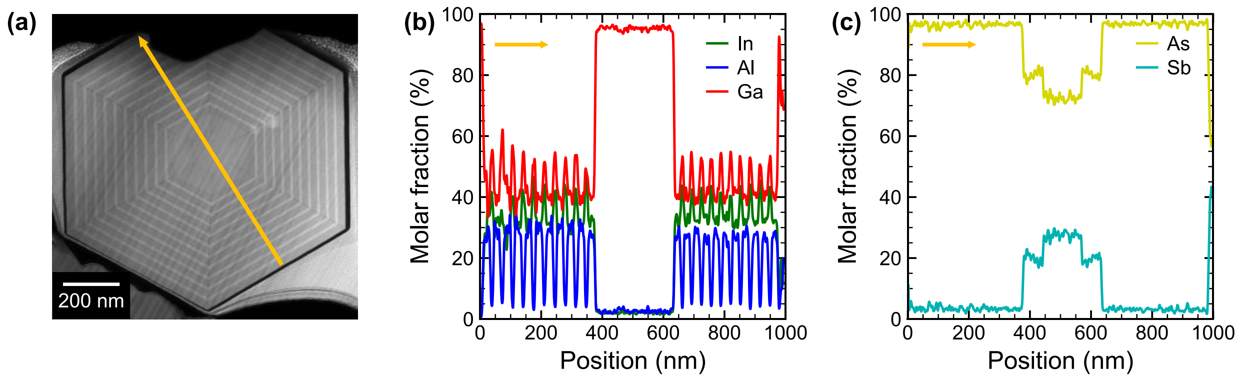

Figure S4: Compositional analysis of the MQW NW-laser heterostructure. (a) HAADF-STEM image in radial cross-section and (b, c) EDXS line scans of group-III and group-V species across the entire NW taken along a $\langle 1\bar{1}0 \rangle$ direction (orange arrow in a).

Figure S5 shows additional high-resolution STEM-HAADF images of the interface regions of the InGaAs QW and adjacent InAlGaAs barrier to illustrate the coherent, defect-free growth. The different images recorded at different sidewall facets of the same InGaAs QW demonstrate a perfectly ordered crystal lattice with no visible defects or dislocations within the MQW region that could be related to strain relaxation. Also, the sharp interfaces between the different layers (InAlGaAs barriers and InGaAs QWs) are clearly resolved, confirming the abruptness and high quality of the grown heterostructures.

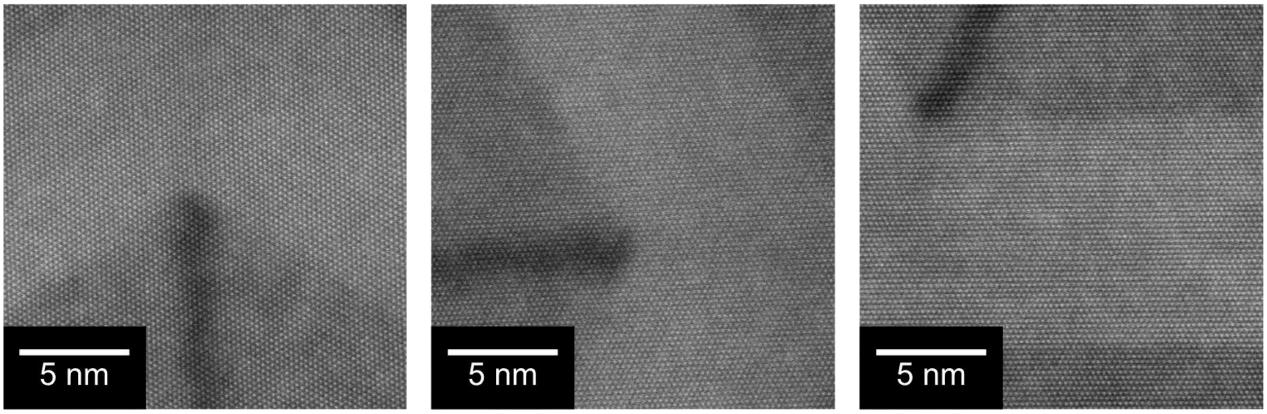

Figure S5: High-angle annular dark-field scanning transmission electron microscopy (STEM-HAADF) images showing three different positions at the interface between the first InGaAs QW and adjacent InAlGaAs barriers. The first two images are taken at corner facets, while the third image shows the interface along one of the six equivalent ⟨110⟩ directions, providing a comprehensive view of the crystalline quality and interface abruptness.

S3. Micro-Photoluminescence (μ-PL) Spectroscopy / Lasing Characteristics

We studied the emission from individual MQW NW-lasers (standing as-grown on the Si substrate) using μ-PL spectroscopy in our home-built setup employing a He-flow cryostat. The individual NWs were characterized under a pulsed excitation scheme using a mode-locked titanium sapphire laser (∼1.59 eV, 200-fs pulses, 82 MHz repetition rate) with a confocal spot size of ∼2 μm. Photoluminescence emission was collected in a head-on geometry from top using a lN_2-cooled InGaAs CCD camera. In comparison to the pump-fluence dependent spectra recorded at 10K in the main text, **Figure S6** shows the spectra and threshold statistics of two additional NWs measured from the same sample (labelled as NW2 and NW3). Like for NW1 (as presented in the main text, Fig. 2), both NW2 and NW3 clearly demonstrate a non-linear increase of a single peak emission at ~1.34 µm (telecom O-band) characteristic of amplified spontaneous emission, which transitions to linear scaling above threshold with increasing pump fluence, whose intensity is

orders of magnitude higher than the spontaneous emission (SE) background, confirming lasing emission. In addition, from corresponding input-output (L-L) characteristics shown in **Figure S6** (b) and (d), both NWs exhibit lasing thresholds of 160 µJ/cm^2 and 209 µJ/cm^2, respectively, which are very similar to the lasing threshold of NW1 presented in the main text.

Inspection of the amplified spontaneous emission (ASE) regime, shown in the highly resolved spectra of **Figure S7**a,c, provides further information of the underlying resonator cavity and mode structure. Here, the presence of distinct Fabry-Perot (FP) oscillations of confined transversal modes in the vertical-cavity NW laser are clearly observed. Particularly useful information can be extracted when closely investigating the spectra of NW3, where two distinct, split sets of FP peaks are best resolved. At around the main lasing peak, two sharp peaks with varying intensities can be seen, which we tentatively attribute to HE-type twin modes given their small energetic splitting (~2-3 meV). Below in FDTD simulations we directly illustrate that it is, indeed, one of two high-order $HE_{4,1,a/b}$ twin modes that is most likely responsible for lasing. Secondly, as illustrated in **Figure S7**b,d the mode sets of Peak1, Peak3, Peak5 (first twin mode) and Peak2, Peak4 (second twin mode) show very similar energetic spacing values around ∼11 meV. For the given NW-cavity length (7.4 µm) this results in a group index (n_g) of 8.38 for our MQW NW-laser using the formula $\Delta E = hc_0/2Ln_g$, where h is Planck's constant, c_0 is vacuum speed of light and ΔE is the energetic spacing of the modes. The high group refractive index reflects the presence of such high-order mode and further explains its strong light-matter interaction in the NW cavity.

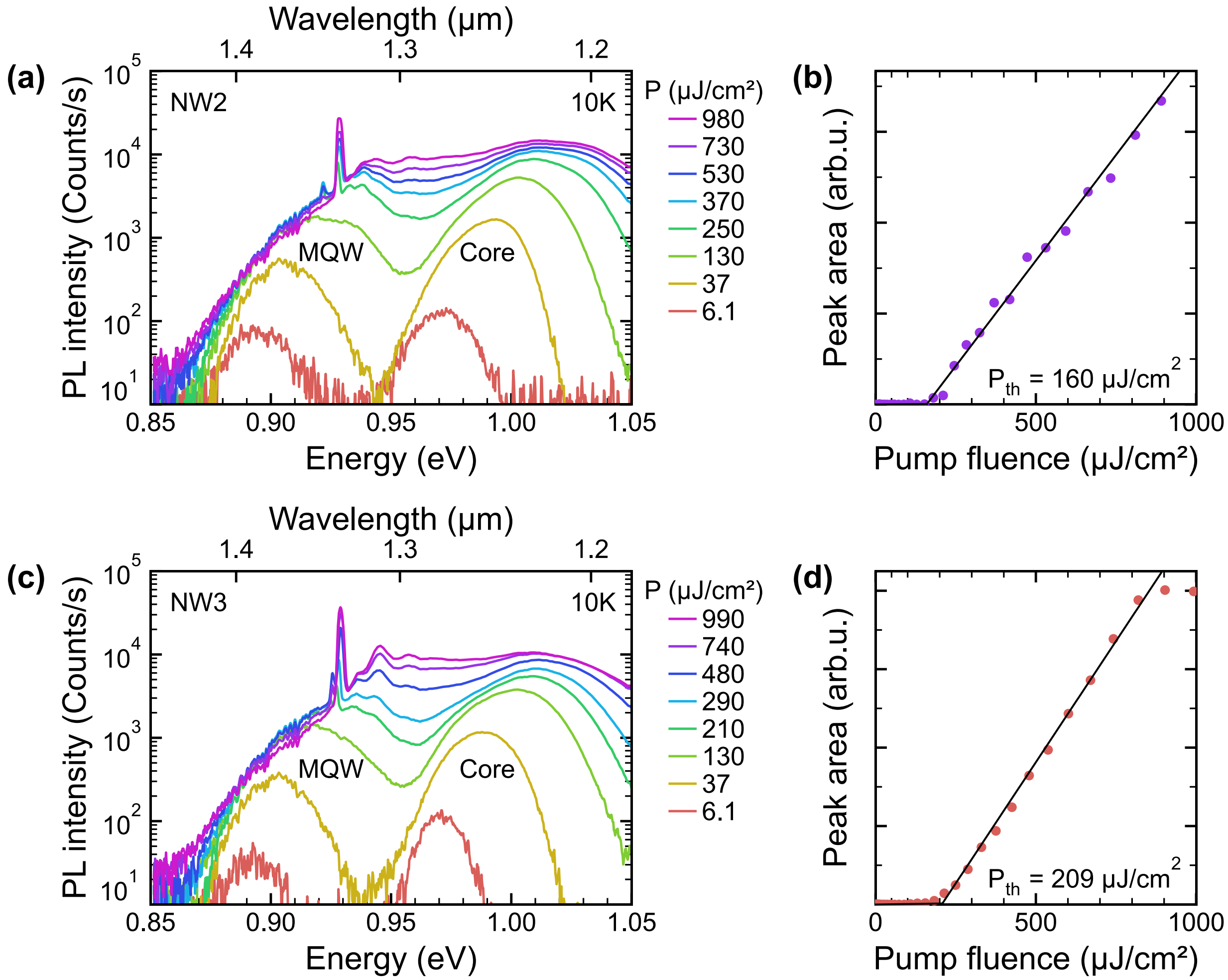

Figure S6: Lasing statistics of MQW NW-lasers on silicon. (a, c) Pump-fluence dependent PL spectra of NW2 and NW3 probed in vertical geometry on Si at 10K. (b, d) Light input – light output (L-L) curves in linear representations showing relatively small variations in lasing threshold.

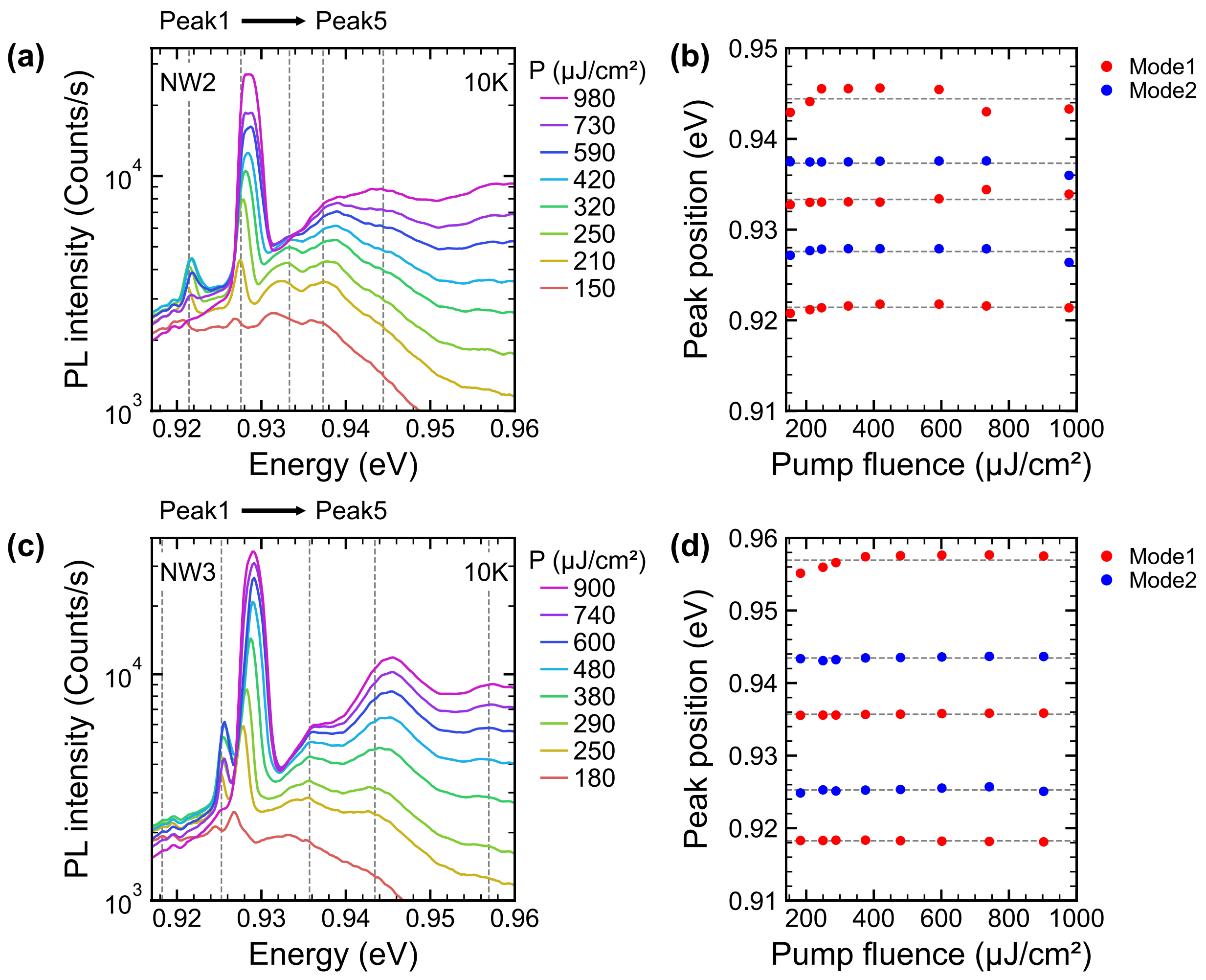

Figure S7: Transition region from ASE to lasing oscillations of different NWs. (a, c) Pump-fluence dependent PL spectra in the region of interest, revealing two distinct sets of Fabry-Perot modulations and corresponding energy spacings (b,d), as taken from grey dashed lines.

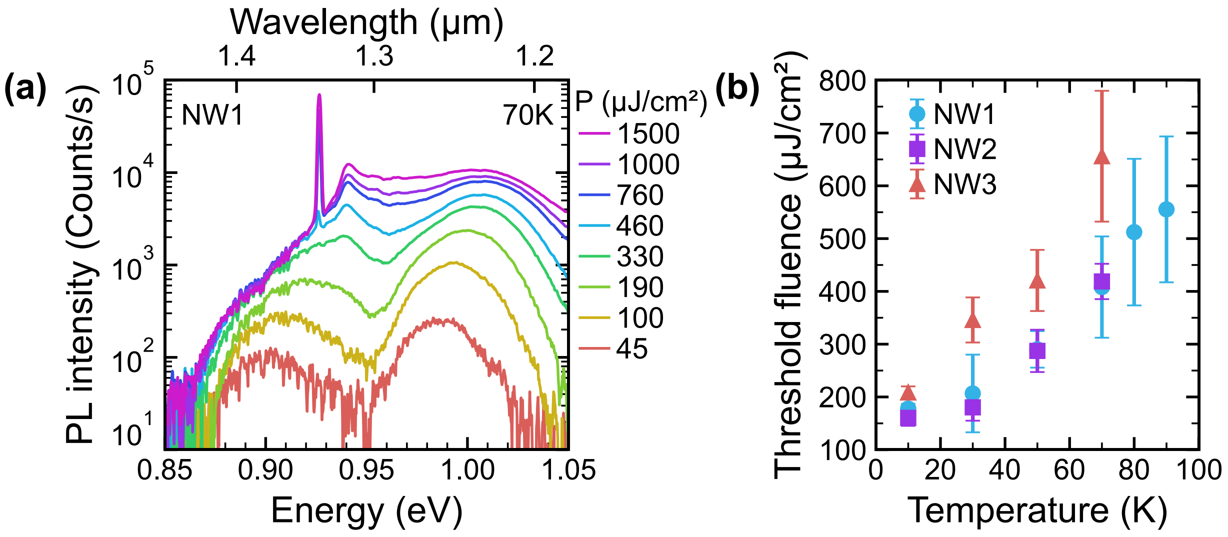

Figure S8: Temperature performance of MQW NW-laser in vertical geometry on Si. (a) Typical pump-fluence dependent PL spectra recorded at 70 K (maximum temperature at which lasing is observed); (b) lasing threshold evolution of all investigated NWs.

Finally, **Figure S8**a shows pump-fluence dependent PL-spectra of the same NW laser presented in the main text (Fig. 2a) at 70K. This is the maximum temperature at which lasing was observed in the as-grown geometry. **Figure S8**b depicts the corresponding evolution of lasing threshold from 10-90K, in comparison with data obtained for the two other NWs (NW2, NW3).

Comparison of Lasing Metrics with other reports

The lasing threshold values that are extracted from the present vertical-cavity NW lasers are put in perspective with other data reported in literature, see **Table 1**. We wish to emphasize that whilst this provides a good overview of some related work, a direct comparison of threshold values has to be treated with care, because different optical pumping schemes, the use of different substrates and in-plane vs. vertical-cavity NW geometries (or the use of NW-array lasers) can all have strong influences on the resulting lasing metrics.

Reference	Material system	Substrate	Measured system	Cavity length (μm)	Wavelength (nm)	Threshold	Operating temperature (K)
This Work	InGaAs - GaAsSb	Si	Vertical - single MQW NW	7.3 ± 0.2	1340	178 μJ/cm^2	70
Schreitmüller et. al. [7].	GaAs(Sb) - AlGaAs	Si	Lying on sapphire – single core-shell NW	7 ± 1	879	53 μJ/cm^2	RT
Temu et. al. [8].	GaAs - InGaAs - InGaP	SOI	Vertical – NW PCSEL array	1.2	966	103 μJ/cm^2	RT
Zhang et. al. [9].	InGaAs - InP	InP	Vertical – MQW NW array	4	1356 - 1542	28.2 μJ/cm^2	RT
Schmiedeke et.al. [10].	InGaAs – (In,Al)GaAs	Si	Lying on sapphire – single MQW NW	12 ± 1	1280	44 μJ/cm^2	RT
Skalsky et. al. [11].	GaAsP - GaAs	Si	Lying on silicon – single MQW NW	10	770	100 μJ/cm^2	RT
Zhang et. al. [12].	InP - InAs	InP	Lying on silicon – single NW	10.5 ± 1.5	1200 - 1600	2.15 mJ/cm^2	RT
Ren et. al. [13].	GaAsSb	Si	Lying on silicon – single NW superlattice	10	890 - 990	75 μJ/cm^2	RT
Schuster et. al. [14].	InGaAs - InP	Si	Vertical – single MQW nano pillar	23.5	1210	36 kW/cm^2	4

Table 1: Comparative overview of key performance metrics for nanowire lasers across different III-V material systems, substrates, and device architectures. The table includes cavity length, lasing wavelength, threshold value, and operating temperature. All values are as reported in the original publications. RT = room temperature (~295 K).

S4. Numerical Simulations of Threshold Gain

The threshold gain of the MQW NW-laser standing on silicon was modelled based on the geometrical and compositional parameters obtained from the cross-sectional STEM analysis in **Figure S4**a and the SEM images presented in Figure1b (main text) and **Figure S9**a. Hence, as input parameters for the numerical simulation, the refractive indices (n) of the MQW NW-laser ($n_{barrier}$=3.22, n_{QW}=3.42, n_{core}=3.53, n_{cap}=3.40) as-grown on Si⟨111⟩ substrate ($n_{substrate}$= 3.50) can be defined, which is anchored via a small hole opening (d_{hole} = 50 nm) in a surrounding 15-nm thick SiO_2 mask layer (n_{mask}=1.46). Also, as seen in **Figure S9**a and schematically depicted in **Figure S9**b, the whole MQW NW structure is surrounded by a ≈1 µm thick parasitic layer ($n_{parasitic}$=3.27) that forms during the shell growth, but which is separated laterally by a 150 nm spacing from the NW. Then, by the combination of finite difference time domain (FDTD) simulations for effective refractive index and end facet reflectivity as well as finite element (FE) simulations for confinement factor, the threshold gain is calculated for varying GaAsSb core diameters at given emission wavelength of 1.34 µm. The variation of core diameter is based on the dimensions found in core-reference samples [1,15]. The threshold gain is, thus, calculated as: $g_{th} = \frac{1}{\Gamma}(\alpha_m + \alpha_i)$, where Γ is the confinement factor, α_m and α_i are mirror and intrinsic losses of the NW cavity. For NW lasers, the intrinsic losses can be neglected, as the threshold is dominated by the end facet reflectivity as $\alpha_m = \frac{1}{2L}\ln\left(\frac{1}{R_1 R_2}\right)$, where the NW length of L is chosen as 7.4 µm and R_1, R_2 are top and bottom facet reflectivity, respectively. In **Figure S10**, the calculated confined optical modes in the MQW NW-laser cavity, along with their confinement factor

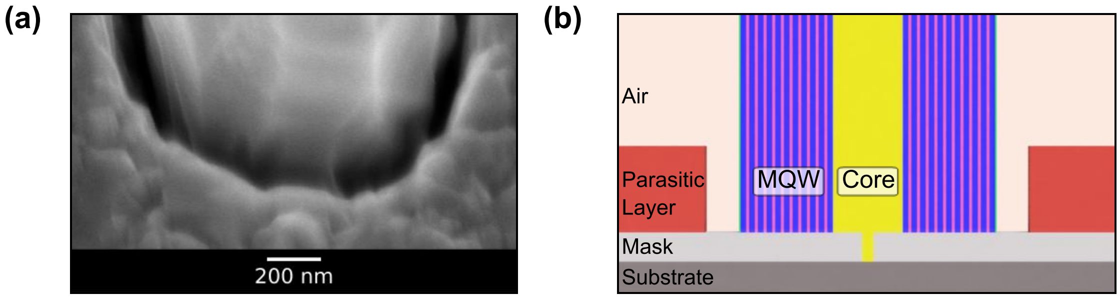

Figure S9. Simulation geometry of the MQW NW laser. (a) SEM image of the stem region of the MQW NW structure, where a 150 nm wide air gap to the adjacent parasitic layer is visible. (b) The simulated geometry depicting the GaAsSb core and surrounding InGaAs/InAlGaAs MQW heterostructure, used for the threshold gain calculations. Here, the parasitic layer (red) is defined as 1 µm according to the corresponding SEM image in (a). The light background color indicates the air surrounding the entire structure, while the light grey and dark grey regions at the bottom refer to the SiO_2 mask layer and Si-substrate.

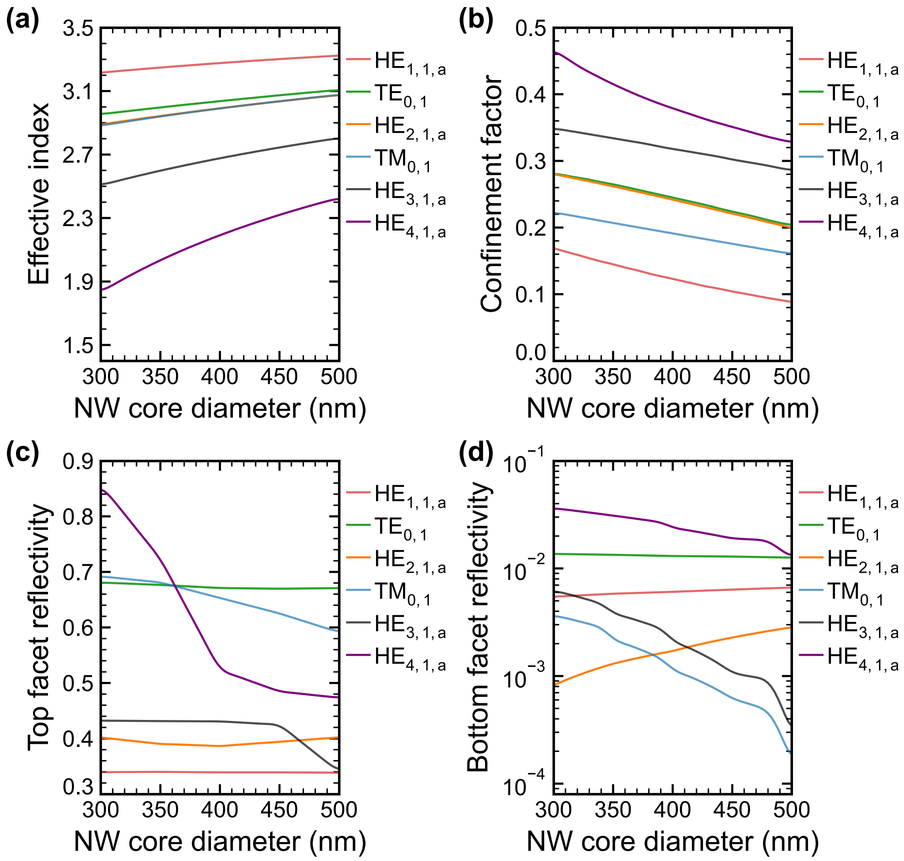

Figure S10. Threshold gain calculation of the MQW NW-laser. (a) effective index, (b) confinement factor, (c) top facet and (d) bottom facet reflectivity of fundamental transverse optical modes, at the given lasing wavelength of 1.34 µm and nanowire length of 10 µm. The NW bottom facet reflectivity is plotted on a semi-logarithmic scale for better illustration.

and reflectivity values can be seen.

While the present design is limited to overall low modal reflectivity at the SiO_2/Si interface, **Figure S11** illustrates how the reflectivity of the anticipated $HE_{4,1,a}$ lasing mode can be improved by increasing the SiO_2 mask thickness from an initial 15 nm to 300 nm. The data shows an order of magnitude increase in modal reflectivity, which is crucial for further reducing the lasing threshold, as it improves optical feedback within the vertical nanowire cavity. The high refractive index contrast between the GaAsSb NW core and the SiO_2 mask layer is key to this effect, enabling strong optical confinement and high-quality cavity modes. Growth of vertical-cavity NW-lasers on Si using thick (>200 nm) SiO_2 mask layers has indeed been demonstrated in our recent work, but requires special strategies in the fabrication of narrow mask openings (e.g. sacrificial NW-template designs) [16].

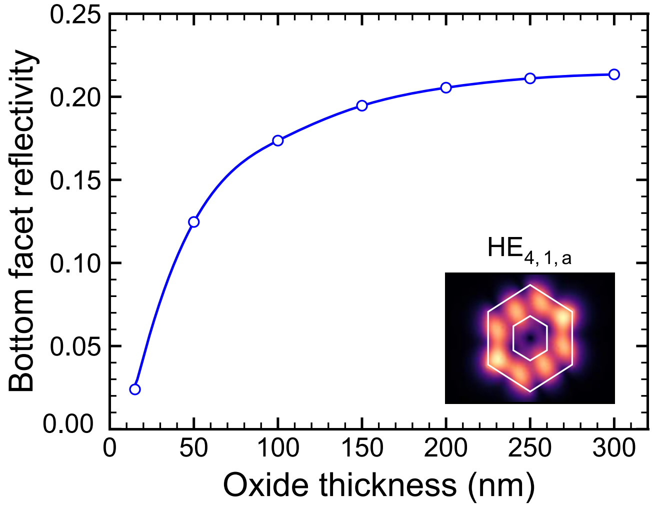

Figure S11. Optimization of the bottom facet modal reflectivity of the anticipated $HE_{4,1,a}$ lasing mode. Increasing the SiO_2 thickness from an initial 15 nm mask to 300 nm, increases the modal reflectivity of lasing mode by about an order of magnitude.

S5. Scanning Transmission Electron Microscopy (STEM)

A STEM cross-section of a single nanowire was prepared perpendicular to the wire axis [111] at approximately half height of the NW with a focused ion beam facility (FEI Helios G3 UC) employing Ga ions. STEM investigations of the cross section were conducted along zone axis [111] at 300 kV with probe-corrected FEI Titan Themis microscope, equipped with a Super-X EDX detector. For Nanobeam Electron Diffraction (NBED) experiments, a probe-semi convergence angle of 2.99 mrad was chosen such that neighboring diffraction orders do not overlap. The STEM beam was scanned across the whole cross-section at a 699×700 raster of scan pixels. A Quantum Detectors Medipix MerlinEM Quad detector was used to record one diffraction pattern at each scan point, resulting in a total of 489300 diffraction patterns. The recording was performed

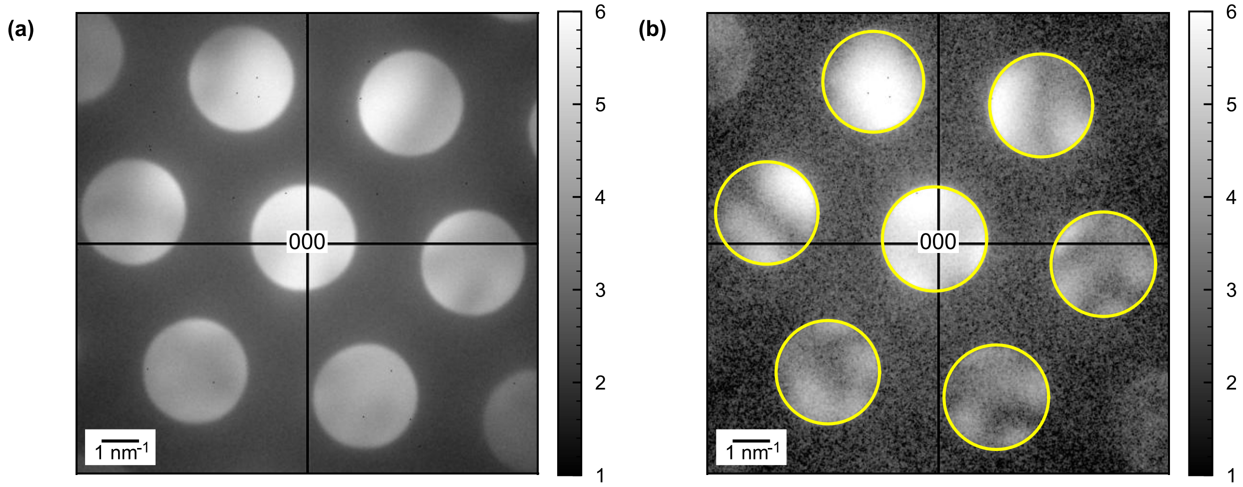

Figure S12. Example diffraction patterns of the 4D STEM experiment. (a) Average over 130 NBED patterns recorder along [111], showing the six $\{20\bar{2}\}$ reflections and the undiffracted beam in the center. The black cross subdivides the detector into four quadrants arises from the 2×2 chip tiling of the Medipix Quad detector. Note the low diffuse background intensity level, indicating negligible thermal diffuse and plasmon scattering, being a prerequisite for precise NBED strain measurements. (b) Representative single NBED pattern. The yellow markers indicate the disc detection result using the radial gradient maximization algorithm. Both diffraction patterns in (a,b) are shown at logarithmic scale, corresponding to the intensity scaling used during disc detection, for which the formula $I_{scaled} = \log\left(1 + 1000 \cdot \frac{I}{\max(I)}\right)$ was used. Note that the coordinate frames of the scan and camera are rotated to each other by approximately 68.5 degrees.

in Single Pixel mode with 12-bit dynamic range, a threshold of 40 keV and a frame time of 1 ms synchronized with the STEM scan. **Figure S12**a,b show representative position-averaged and single NBED diffraction patterns at logarithmic intensity scale.

Measurement of Local Lattice Variations by Nano-Beam Electron Diffraction (NBED)

The positions of the six $\{20\bar{2}\}$ Bragg discs available in [111] zone axis geometry and of the undiffracted beam were determined with subpixel accuracy by Radial Gradient Maximization [17]. The three $\{20\bar{2}\}$ Friedel pairs are parallel to three major radial growth directions $\langle 10\bar{1}\rangle$ of the NW perpendicular to the layer facets. Hence, the projections of the measured position vectors of the reflections onto $\langle 10\bar{1}\rangle$ directly correspond to lattice plane variations in the respective radial growth directions. In the main article, the variations of the reflection positions have been projected onto the directions $[20\bar{2}]$ and $[1\bar{2}1]$. These are perpendicular to each other and directly show the relative lattice parameter along growth direction and along one in-plane direction for the two horizontally aligned sectors of the hexagonal cross-section of the NW. Because the growth directions within the other sectors are rotated by $\pm 60°$ and $\pm 120°$ with respect to $[20\bar{2}]$, variations of both κ_{xx} and κ_{yy} are seen in these sectors in Figure 4a,b. Note that Figure 4 contains the complete information about the relative lattice spacings in all sectors.

However, the lattice variation parallel to all six radial growth facets is particularly interesting as it provides information about possible coherent intergrowth or lattice relaxation at the interface due the defects. Therefore, to demonstrate fully-strained growth such that the lattice parameters vary only along the growth directions $[20\bar{2}]$, $[2\bar{2}0]$ and $[0\bar{2}2]$, we additionally present the evaluations with the definitions $\kappa_{xx} = \frac{d_{2\bar{2}0}}{d^0_{2\bar{2}0}}$, $\kappa_{yy} = \frac{d_{11\bar{2}}}{d^0_{11\bar{2}}}$ in **Figure S13**, and with $\kappa_{xx} = \frac{d_{0\bar{2}2}}{d^0_{0\bar{2}2}}$, $\kappa_{yy} =$

$\frac{d_{2\bar{1}\bar{1}}}{d^0_{2\bar{1}\bar{1}}}$ in **Figure S14**. All the resulting maps for κ_{xx} and κ_{yy} were obtained by averaging over the lattice variation calculated from all individual reflections, weighted according to the respective signal-to-noise ratios derived from the summed reflection intensity and the background level.

In agreement with **Figure 4**, **Figure S13** and **Figure S14** reveal a radial lattice expansion in the quantum wells and a radial lattice contraction in the outer core within the respective sector pairs. Moreover, the lattice variation parallel to the radial growth facets is small and free of abrupt changes at interfaces between the layers. Also the line profiles confirm the observations described above and are in quantitative agreement with **Figure 4**c,d in the main article, respectively.

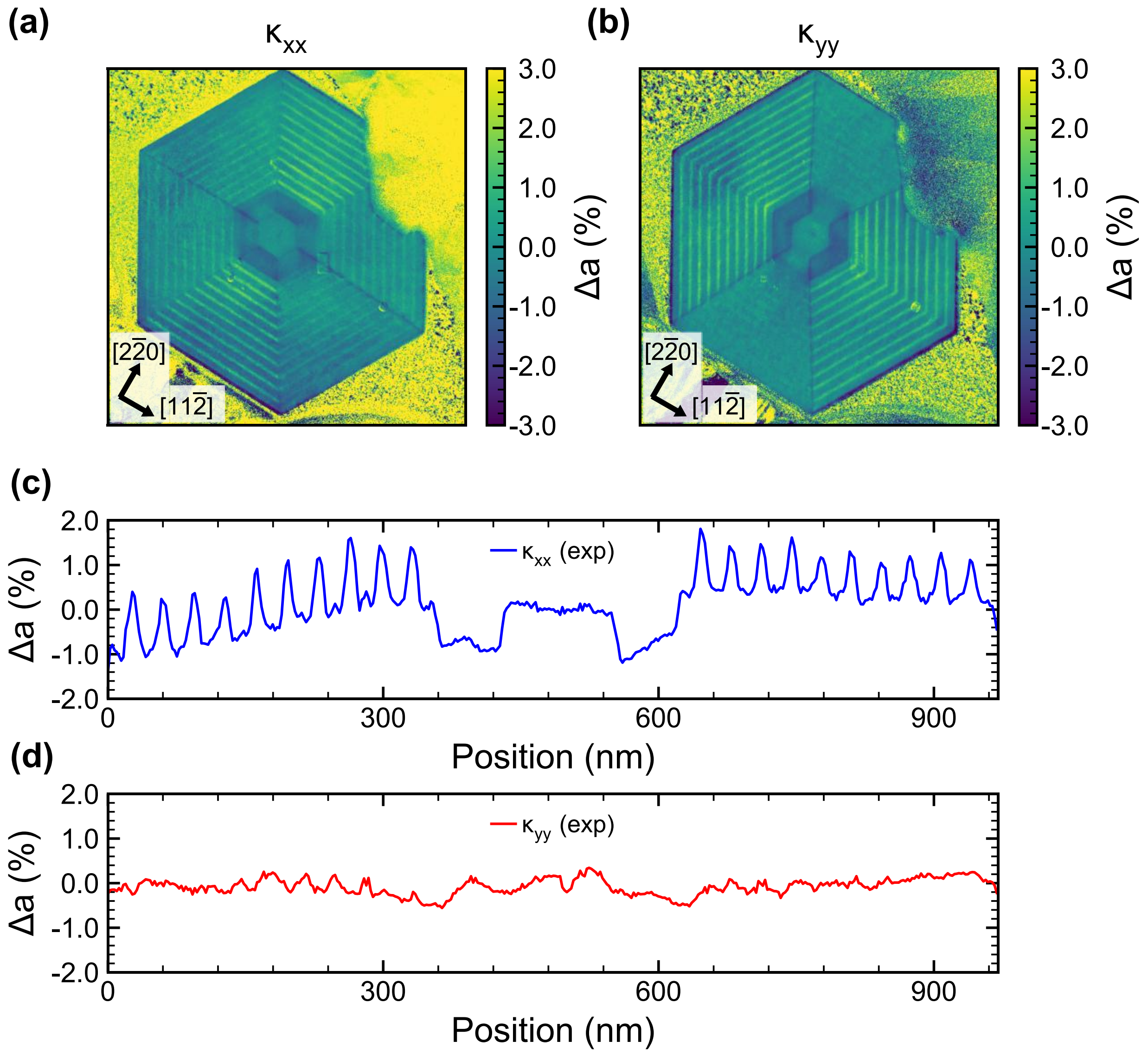

Figure S13. Relative lattice parameter evaluation with basis setting $\kappa_{xx} = \frac{d_{2\bar{2}0}}{d^0_{2\bar{2}0}}$, $\kappa_{yy} = \frac{d_{11\bar{2}}}{d^0_{11\bar{2}}}$. In quantitative agreement with Figure 4 of the main article, fully strained growth is observed with nearly constant in-plane lattice parameters and a lattice parameter variation along growth direction as for the horizontal sectors.

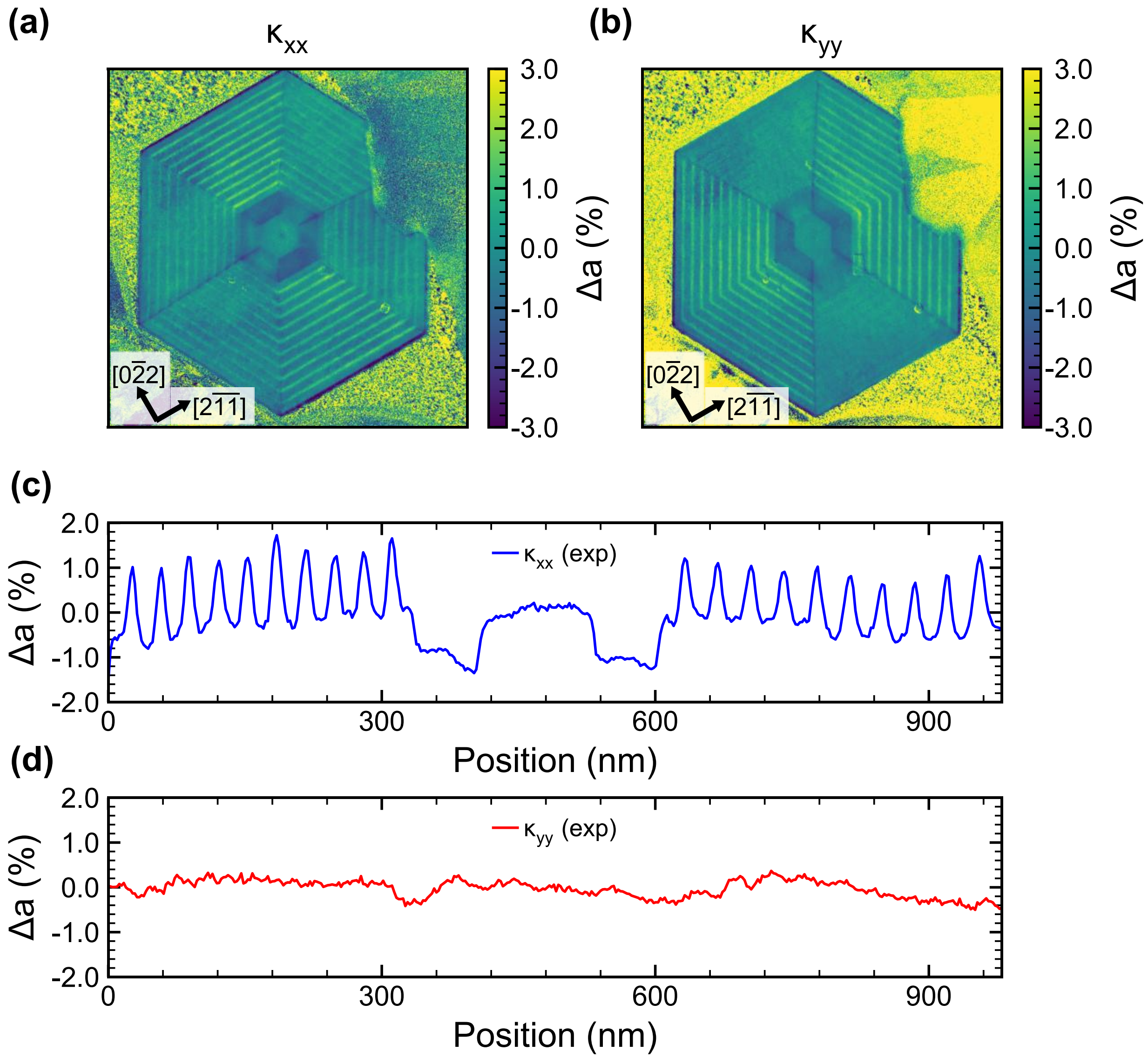

Figure S14. Relative lattice parameter evaluation with basis setting $\kappa_{xx} = \frac{d_{0\bar{2}2}}{d^0_{0\bar{2}2}}$, $\kappa_{yy} = \frac{d_{2\bar{1}\bar{1}}}{d^0_{2\bar{1}\bar{1}}}$. In quantitative agreement with Figure 4 of the main article, fully strained growth is observed with nearly constant in-plane lattice parameters and a lattice parameter variation along growth direction as for the horizontal sectors.

S6. Modelling of Strain

The numerical calculations of the strain in the MQW NW heterostructure were carried out using the nextnano-3 software [10]. In **Figure S15** the simulated lateral (ε_{xx}) and vertical (ε_{yy}) strain components are shown, which estimates the QWs to be tensile strained (ε_{xx}~ 1.6%) in the lateral direction. This is further supported by the 2D-map of the elastic energy density shown in **Figure S16**a for the MQW NW heterostructure at 10K. This illustrates that the QWs and VS-grown region of the GaAsSb core are strained, whereas the quaternary barriers and VLS-grown GaAsSb core are strain-free. Also, the resulting band profile of the entire heterostructure is shown in **Figure S16**b under the influence of strain at 10K, as taken along the radial $\langle 1\bar{1}0 \rangle$ direction (indicated by an orange arrow in **Figure S16**a). The band-edges of the Γ conduction band and heavy hole valance band are depicted in blue and red data, respectively.

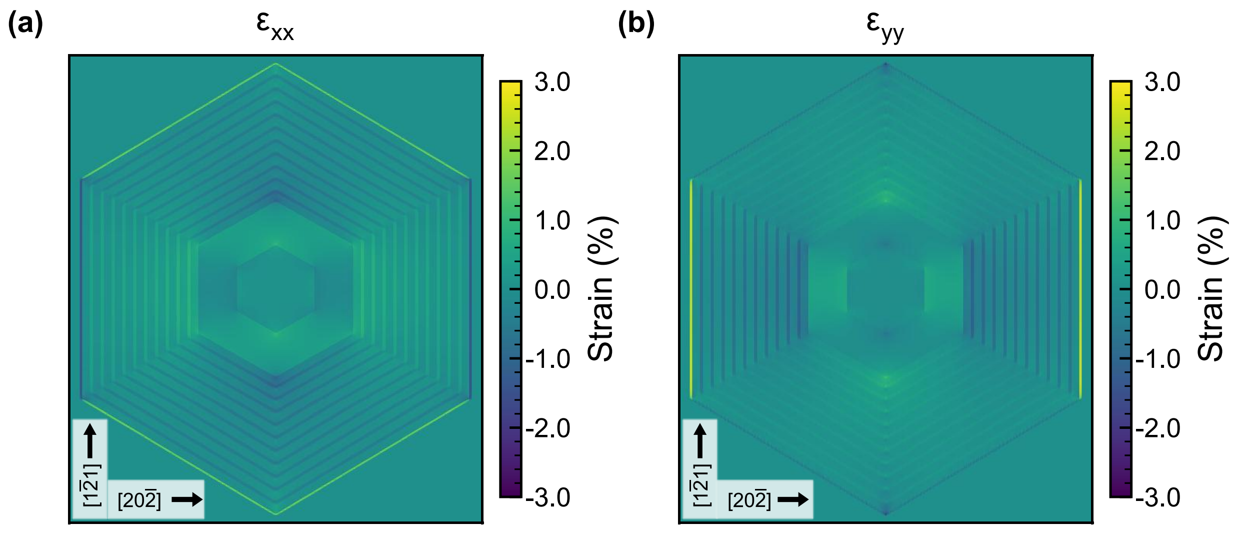

Figure S15. Simulated strain maps of the NW-laser heterostructure. (a,b) 2D-maps of the radial strain distribution of the ε_{xx} and ε_{yy} components, respectively.

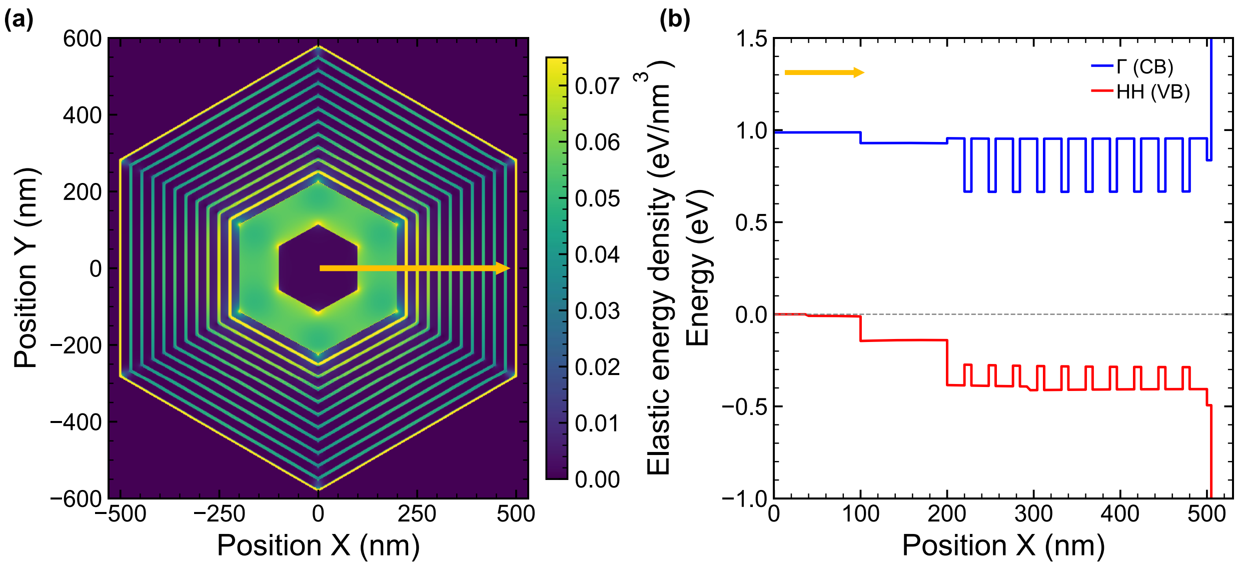

Figure S16. Calculated elastic energy density of the MQW NW heterostructure, and associated electronic band profile. (a) 2D-map of the elastic energy density of MQW NW laser at 10K. GaAs outer cap is masked for better visualization. (b) Radial profile of the corresponding band profile (conduction band – blue, valence band – red) under the given strain, as taken along the orange arrow in (a) at 10K. The grey dashed line shows the Fermi-level (E_F) set to be zero.

REFERENCES

[1] Schmiedeke, P.; Döblinger, M.; Meinhold-Heerlein, M.-A.; Doganlar, C.; Finley, J. J.; Koblmüller, G. Sb-saturated high-temperature growth of extended, self-catalyzed GaAsSb nanowires on silicon with high quality. *Nanotechnol.* **2023**, 35, 055601.

[2] Plissard, S.; Larrieu, G.; Wallart, X.; Caroff, P. High yield of self-catalyzed GaAs nanowire arrays grown on silicon via gallium droplet positioning. *Nanotechnol.* **2011**, 22, 275602.

[3] Munshi, A. M.; Dheeraj, D. L.; Fauske, V. T.; Kim, D. C.; Huh, J.; Reinertsen, J. F.; Ahtapodov, L.; Lee, K. D.; Heidari, B.; van Helvoort, A. T. J.; Fimland, B. O.; Weman, H. Position-controlled uniform GaAs nanowires on silicon using nanoimprint lithography. *Nano Lett.* **2014**, 14, 960–966.

[4] Russo-Averchi, E.; Plestina, J. V.; Tütüncüoglu, G.; Matteini, F.; Dalmau-Mallorquí, A.; de la Mata, M.; Rüffer, D.; Potts, H. A.; Arbiol, J.; Conesa-Boj, S.; i Morral, A. F. High yield of GaAs nanowire arrays on Si mediated by the pinning and contact angle of Ga. *Nano Lett.* **2015**, 15, 2869–2874.

[5] Vukajlovic-Plestina, J.; Kim, W.; Ghisalberti, L.; Varnavides, G.; Tütüncuoglu, G.; Potts, H.; Friedl, M.; Güniat, L.; Carter, W. C.; Dubrovskii, V. G.; i Morral, A. F. Fundamental aspects to localize self-catalyzed III-V nanowires on silicon. *Nature Comm.* **2019**, 10, 2281.

[6] Uccelli, E.; Arbiol, J.; Magen, C.; Krogstrup, P.; Russo-Averchi, E.; Heiss, M.; Mugny, G.; Morier-Genoud, F.; Nygård, J.; Morante, J.; Fontcuberta I Morral, A. Three-dimensional multiple-order twinning of self-catalyzed GaAs nanowires on Si substrates. *Nano Lett.* **2011**, 11, 3827–3832.

[7] Schreitmüller, T.; Jeong, H. W.; Esmaielpour, H.; Mead, C. E.; Ramsteiner, M.; Schmiedeke, P.; Thurn, A.; Ajay, A.; Matich, S.; Döblinger, M.; Lauhon, L. J.; Finley, J. J.; Koblmüller, G. Large Tolerance of Lasing Properties to Impurity Defects in GaAs(Sb)-AlGaAs Core-Shell Nanowire Lasers. *Adv. Funct. Mater.* **2024**, 34, 2311210.

[8] Temu, B.; Yan, Z.; Ratiu, B.-P.; Oh, S. S.; Li, Q. Room temperature lasing from InGaAs quantum well nanowires on silicon-on-insulator substrates. *Appl. Phys. Lett.* **2024**, 125, 223501.

[9] Zhang, X.; Zhang, F.; Yi, R.; Wang, N.; Su, Z.; Zhang, M.; Zhao, B.; Li, Z.; Qu, J.; Cairney, J. M.; Lu, Y.; Zhao, J.; Gan, X.; Tan, H. H.; Jagadish, C.; Fu, L. Telecom-band multiwavelength vertical emitting quantum well nanowire laser arrays. *Light Sci. Appl.* **2024**, 13, 230.

[10] Schmiedeke, P.; Thurn, A.; Matich, S.; Döblinger, M.; Finley, J. J.; Koblmüller, G. Low-threshold strain-compensated InGaAs/(In,Al)GaAs multi-quantum well nanowire lasers emitting near 1.3 µm at room temperature. *Appl. Phys. Lett.* **2021**, 118, 221103.

[11] Skalsky, S.; Zhang, Y.; Alanis, J. A.; Fonseka, H. A.; Sanchez, A. M.; Liu, H.; Parkinson, P. Heterostructure and Q-factor engineering for low-threshold and persistent nanowire lasing. *Light Sci. Appl.* **2020**, 9, 43.

[12] Zhang, G.; Takiguchi, M.; Tateno, K.; Tawara, T.; Notomi, M.; Gotoh, H. Telecom-band lasing in single InP/InAs heterostructure nanowires at room temperature. *Sci. Adv.* **2019**, 5, eaat8896.

[13] Ren, D.; Ahtapodov, L.; Nilsen, J. S.; Yang, J.; Gustafsson, A.; Huh, J.; Conibeer, G. J.; van Helvoort, A. T. J.; Fimland, B.-O.; Weman, H. Single-Mode Near-Infrared Lasing in a GaAsSb-Based Nanowire Superlattice at Room Temperature. *Nano Lett.* **2018**, 18, 2304-2310.

[14] Schuster, F.; Kapraun, J.; Malheiros-Silveira, G. N.; Deshpande, S.; Chang-Hasnain, C. J. Site-controlled growth of monolithic InGaAs/InP quantum well nanopillar lasers on silicon. *Nano Lett.* **2017**, 17, 2697-2702.

[15] Schmiedeke, P.; Doganlar, C.; Jeong, H. W.; Döblinger, M.; Finley, J. J.; Koblmüller, G. Low-threshold single ternary GaAsSb nanowire lasers emitting at silicon transparent wavelengths. *Appl. Phys. Lett.* **2024**, 124, 071112.

[16] Koblmüller, G.; Mayer, B.; Stettner, T.; Abstreiter, G.; Finley, J. J. GaAs-AlGaAs core-shell nanowire lasers on silicon: invited review. *Semicond. Sci. Technol.* **2017**, 32, 053001.

[17] Müller, K.; Rosenauer, A.; Schowalter, M.; Zweck, J.; Fritz, R.; Volz, K. Strain measurement in semiconductor heterostructures by scanning transmission electron microscopy. *Microsc. Microanal.* **2012**, 18, 995-1009.
